# Hydroclimatic changes on multiple timescales since 7800 y BP in the winter precipitation–dominated Central Asia

**DOI:** 10.1073/pnas.2321645121

**Published:** 2024-03-25

**Authors:** Liangcheng Tan, Hai Cheng, Dong Li, Rustam Orozbaev, Yanzhen Li, Hai Xu, R. Lawrence Edwards, Yougui Song, Le Ma, Fangyuan Lin, Ashish Sinha, Zhisheng An

**Affiliations:** ^a^State Key Laboratory of Loess, Institute of Earth Environment, Chinese Academy of Sciences, Xi’an 710061, China; ^b^Institute of Global Environmental Change, Xi’an Jiaotong University, Xi’an 710054, China; ^c^Library of Chang’an University, Xi’an 710064, China; ^d^Research Center for Ecology and Environment of Central Asia (Bishkek), Chinese Academy of Sciences, Bishkek 720040, Kyrgyzstan; ^e^Institute of Geology, National Academy of Sciences of Kyrgyz Republic, Bishkek 720040, Kyrgyzstan; ^f^Institute of Surface-Earth System Science, Tianjin University, Tianjin 300072, China; ^g^Department of Earth and Environmental Sciences, University of Minnesota, Minneapolis, MN 55455; ^h^Department of Earth Science, California State University, Dominguez Hills, Carson, CA 90747; ^i^Interdisciplinary Research Center of Earth Science Frontier, Beijing Normal University, Beijing 100875, China

**Keywords:** westerlies, stalagmite, Holocene, winter insolation, Central Asia

## Abstract

Central Asia (CA) is one of the world’s most significant arid regions, which is markedly impacted by global warming. A better understanding of the dynamical processes governing its Holocene climate variability is critical for a better understanding of possible future impacts of climate change in the region. To date, most of the existing CA paleoclimate records are from the summer precipitation–dominated eastern CA (ECA), with few records from the winter precipitation–dominated western CA (WCA). Here, we present a precisely dated (~6‰) and highly resolved (<4-y) record of hydroclimatic variations from the WCA covering the period between 7,774 and 656 y BP. Utilizing multiple proxies (δ^18^O, δ^13^C, and Sr/Ca) derived from a stalagmite from the Fergana Valley, Kyrgyzstan, we reveal a long-term drying trend in WCA, which is in contrast with the wetting trend in ECA. We propose that different responses of winter and summer westerly jets to seasonal solar insolation over the past 8,000 y may have resulted in an antiphased precipitation relationship between the WCA and ECA. Our data contain dominant quasiperiodicities of 1,400, 50 to 70, and 20 to 30 y, indicating close connections between the WCA climate and the North Atlantic. We further identified a series of droughts and pluvials on centennial-to-decadal timescales, which may have influenced regional societies and trans-Eurasian culture exchanges during historical and prehistorical times.

## Significance

While numerous studies show a moistening trend in ECA over the Holocene, the hydroclimate changes in WCA have remained elusive. In this study, we used various stalagmite-based geochemical proxies from Kyrgyzstan to reconstruct a precise history of hydroclimatic changes from the winter precipitation–dominated WCA over the last 7,800 y. Our data unveil a prolonged drying trend in WCA, superimposed by a series of droughts and pluvials. We attribute divergent precipitation trends in WCA and ECA to disparate responses of winter and summer westerly jets to seasonal solar insolation. Our findings not only deepen our understanding of regional hydroclimate dynamics but also hold the potential to refine future climate change projections in this environmentally fragile region.

Arid Central Asia (CA), spanning across 30° of longitudes and 15° of latitudes, is facing increasing water resource pressure under the impacts of global warming and other human activities. Rates of glacial retreat in the region are accelerating, and ~20% of the glaciers have already disappeared during the past half-century (1, 2). Lake surface areas have also decreased by ~50% over the past 30 y (3). Due to a large number of transboundary rivers and uneven spatial distribution of water resources in this region, the contradictions between water supply and demand not only affect the local ecological environment and socioeconomic development but have also increased the possibility of international conflicts (4).

Holocene climate records provide important constraints for a better understanding of future climate change. Chen et al. (5) suggested a persistent wetting trend in CA, with the wettest period in the late Holocene, as revealed by multiproxy analyses of loess-paleosol sequences in Xinjiang, China. This finding is consistent with the synthesized pollen records from northern Xinjiang and is well supported by other records of eolian deposition (6), peat and lake sediments (7–12), and stalagmites (13) from Xinjiang, China. However, most of the Holocene hydroclimate records in CA are from ECA (14), with their precipitation maximum in the summer half-year. This seasonal precipitation pattern is different from that in WCA, west Asia, and the Mediterranean, where rainfall is concentrated in the winter half-year (15). A recent stalagmite-based study suggested increased and decreased precipitation in WCA during the Medieval Warm Period and the Little Ice Age, respectively (16), which is opposite to the drier Medieval Warm Period and wetter Little Ice Age in ECA (17). Leroy et al. (18) reported a sea level maximum in the southeast corner of the Caspian Sea (CS) during the early–middle Holocene. The water flow gradient was from south CS to north CS during the mid-Holocene, which reversed in the late Holocene (19), implying a drying trend in the WCA during the Holocene. Nevertheless, the driving forces of CS lake level changes during the Holocene are complicated because of the impacts of evaporation, meltwaters from high-altitude glaciers, and supplemental waters from several rivers. It remains unclear whether the hydroclimate changes across CA were synchronous. Highly resolved climate records with accurate chronologic controls from WCA are therefore needed to better understand the hydroclimate patterns of the westerlies-dominated Asia (14).

In this study, we present an accurately dated (~6‰) and highly resolved (<4-y) precipitation record for WCA since 7,800 y BP by integrating multiple geochemical proxies (δ^18^O, δ^13^C, and Sr/Ca ratios) in a stalagmite from Kyrgyzstan. Our reconstruction, together with the existing regional proxy records, reveals orbitally driven contrasting precipitation trends between the WCA and ECA since the middle Holocene. Our results confirm model simulations of increasing precipitation in the winter-precipitation domain during periods with perihelion at the northern hemisphere summer solstice and aphelion at the northern hemisphere winter solstice (15).

## Results

### Stalagmite Proxy Records.

**Fig. 1.**
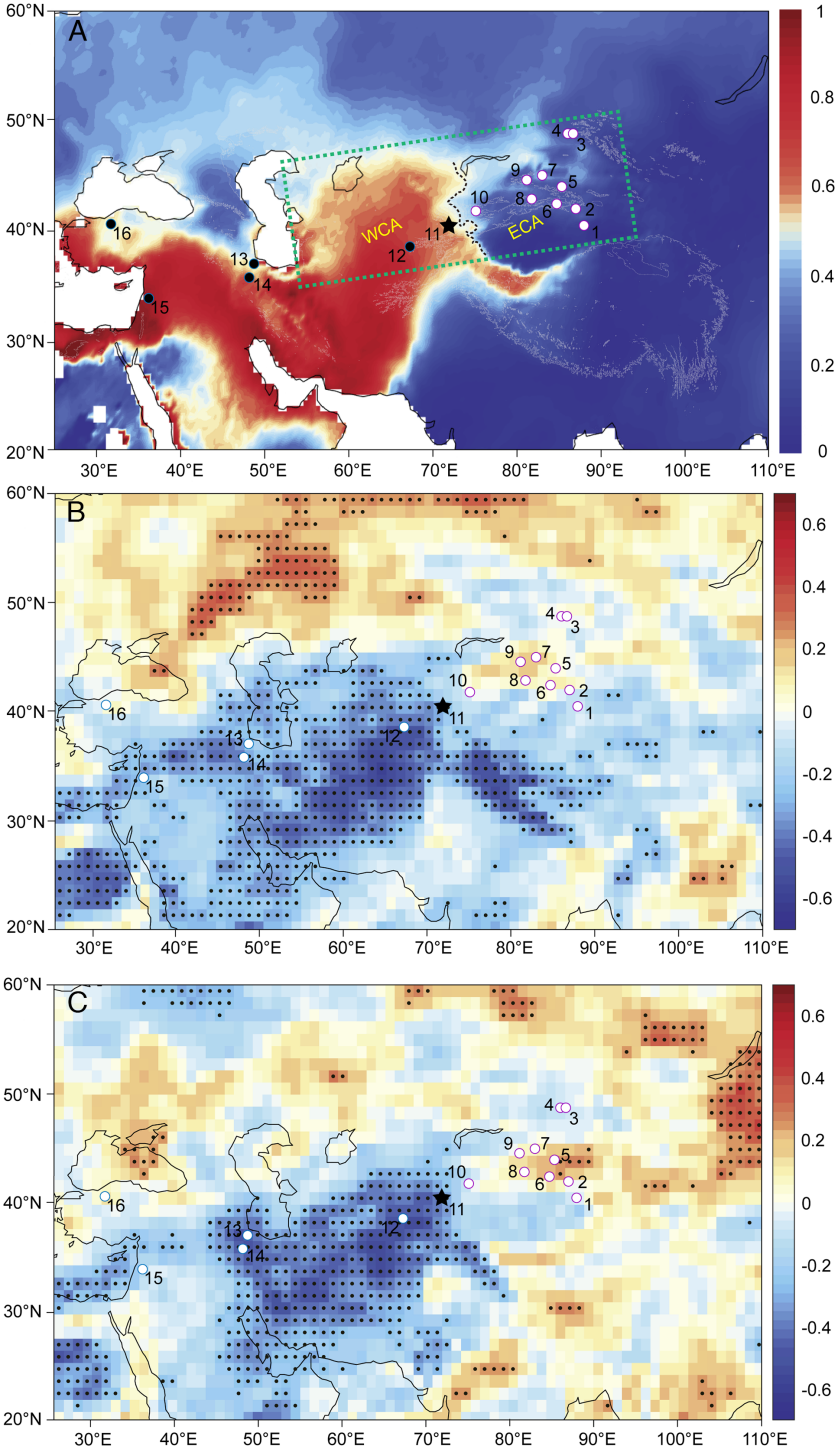
(*A*) Locations of records and the mean ratio of the winter-half-year (November to April) precipitation to annual precipitation in the region from 1950 to 2015. The precipitation data are from the Climate Research Unit (http://www.cru.uea.ac.uk/data), and the topographic data are from the Shuttle Radar Topography Mission digital elevation dataset. (https://cgiarcsi.community/data/srtm-90m-digital-elevation-database-v4-1/). The area enclosed by the green dashed line roughly denotes the location of arid CA. The black dotted line marks today’s boundary between the winter precipitation–dominated WCA and summer precipitation–dominated ECA. (*B* and *C*) are correlations between ECHAM5-wiso simulated δ^18^O_p_ from the grid point closest to Talisman Cave and regional precipitation amount for 1960 to 2015 AD. (*B*) the amount-weighted mean of cold season (Nov.-Apr.) δ^18^O_p_ and precipitation amount, (*C*) amount-weighted annual mean δ^18^O_p_ and precipitation amount. Stippling indicates regions of significant correlations at a 95% significance level. The star denotes Talisman cave (11), and other sites referred to in the text are denoted by solid circles. 1, Chaiwopu peat (7); 2 Lake Bosten (22); 3, Tielishahan peat (23); 4, Narenxia peat (9); 5, Lujiaowan loess (5); 6, Baluk cave (13); 7, Lake Aibi (24); 8, Kesang cave (25, 26); 9, Lake Sayram (27); 10, Lake Son Kol (28); 12, Ton cave (26); 13, Lake Neor (29); 14, Katalekhor cave (30); 15, Jeita cave (31); and 16, Sofular cave (32) (see *SI Appendix*, Table S1 for detailed locations).

**Fig. 2.**
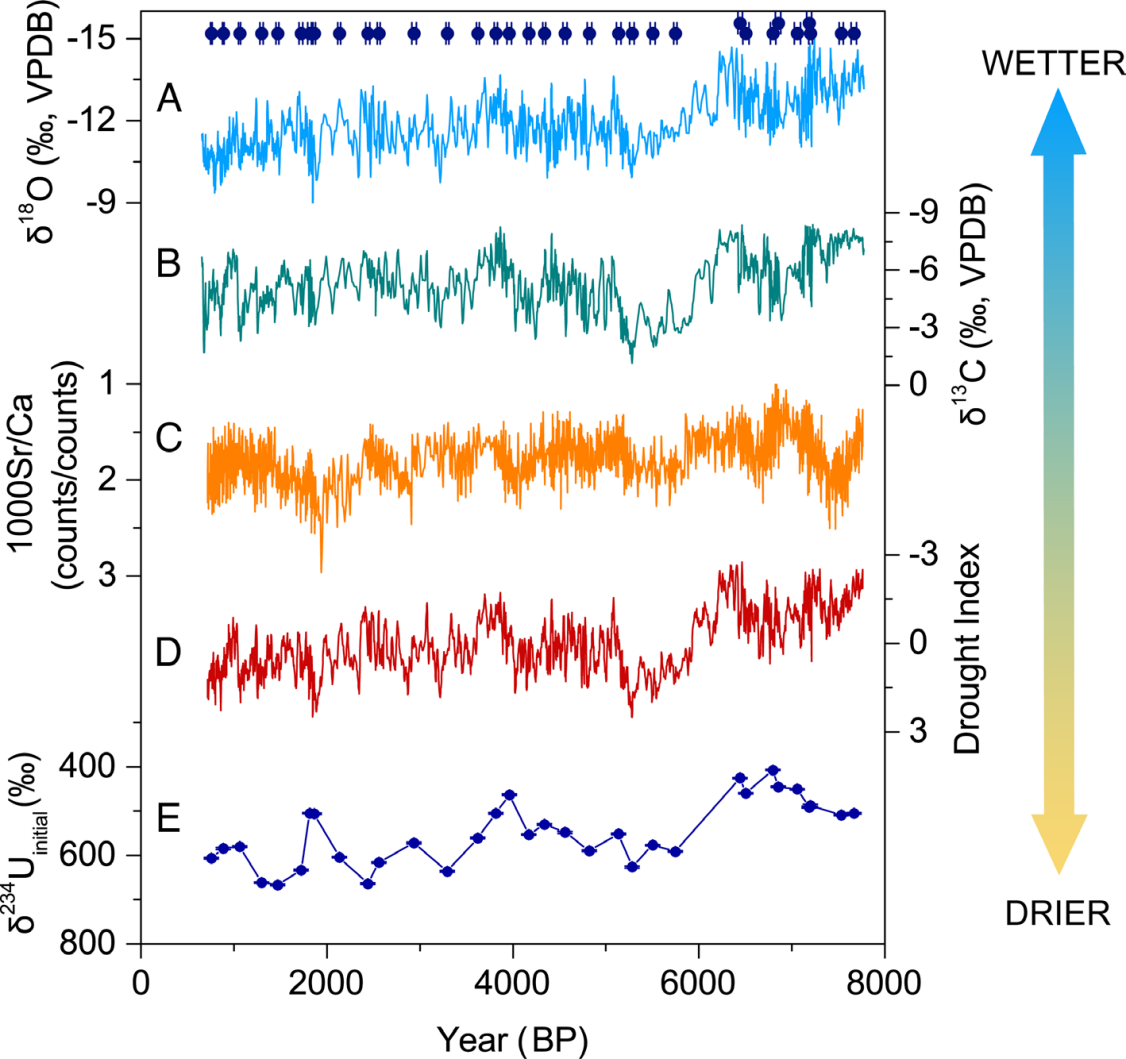
Proxy records of stalagmite F11. (*A*) δ^18^O record; (*B*) δ^13^C record; (*C*) Sr/Ca record; (*D*) drought index record represented by the PCA results of δ^18^O, δ^13^C and Sr/Ca record; (*E*) δ^234^U_initial_ record. Blue dots with error bars represent ^230^Th dates.

Talisman cave is located in the southeastern Fergana Valley in Kyrgyzstan, CA (40.39°N, 72.35°E, 1,486 m a.s.l). Mean annual precipitation in this region is approximately 300 mm, with over 70% being attributed to the rainfall and snowfall during winter and spring (Fig. 1 and *SI Appendix*, Fig. S1). Today, precipitation variability in this region is modulated by changes in the westerly jet (WJ) (20). Two calcite stalagmites, F2 and F11 (*SI Appendix*, Figs. S2 and S3), were collected from the cave in 2015. U-Th dating results (*Materials and Methods*) indicate that F11 was continuously deposited between 7,774 and 656 y BP (year B.P., where “present” means AD 1950), and F2 was deposited from 4,943 to 211 y BP, with a >300-y hiatus between 3,628 and 3,312 y BP (*SI Appendix*, Table S2 and Fig. S4). The average dating uncertainties of F11 are ~6‰. The dating errors in F2 are larger, with a mean error of ~7% for the last 2,000 y and ~1.5% before 2,000 y BP. The δ^18^O and δ^13^C records of the two stalagmites replicate well during the overlapping period (*SI Appendix*, Fig. S5), indicating their deposition occurred at or near isotopic equilibrium fractionation conditions (21). Furthermore, “Hendy test” results show consistent variations of the δ^18^O and δ^13^C values from center to margin in four layers of F11, respectively, which exclude any large kinetic disequilibrium effects during their depositions (*SI Appendix*, Fig. S6). Here, we focus on the records of F11 because the sample quality is much better than F2, with continuous deposition since the middle Holocene, more accurate age control, and higher temporal resolution (~3.6-y). The δ^18^O, δ^13^C, and Sr/Ca records from Talisman cave show an increasing trend with notable centennial- to decadal-scale variations since 7,800 y BP (Fig. 2).

### Drought Index Record.

Significant positive correlations are observed between the δ^18^O, δ^13^C, and Sr/Ca ratios of F11 (*SI Appendix*, Table S3), suggesting local precipitation as their common controlling factor (20). Decreased (Increased) precipitation could reduce (enhance) the vegetation cover/density and soil microbial activity, increase (decrease) the water–rock interaction time and prior calcite precipitation (PCP) in the epikarst, as well as the CO_2_ degassing of drip-water, resulting in more positive (negative) δ^13^C values in the speleothem. Due to the preferential loss of Ca^2+^ from solution, enhanced PCP during dry conditions could also increase the Sr/Ca ratio in the speleothem, although lower growth rates might reduce the Sr/Ca ratio at the same time (33–36). Meanwhile, less (more) snowfall/rainfall during winter and spring, which is characterized by more negative δ^18^O (δ^18^O_p_), will result in higher (lower) weighted mean δ^18^O_p_ values and cause enriched (depleted) δ^18^O values in speleothems in this region (20, 37). Consequently, we applied a principal components analysis method (PCA) to the δ^13^C, δ^18^O, and Sr/Ca records of F11 to extract their common variance. The principal component 1 (PC1), which explains 61% of the total variance, was used as a drought index for this region (*SI Appendix*, Table S4), with higher values representing drier conditions and lower values representing wetter conditions.

The reconstructed drought index record shows good similarities with the initial uranium isotope activity ratios, (^234^U/^238^U)_I_ (also demonstrated as δ^234^U_initial_) of F11, with a lower drought index corresponding to lower δ^234^U_initial_ value and vice versa (Fig. 2), confirming it as a proxy of paleohydrological changes (32, 36, 38). During wetter conditions, faster percolation rates could cause greater dissolution rates, thus enhancing the weathering and dissolution of the carbonate rock, resulting in preferential leaching of ^234^U from crystal lattice sites disrupted by alpha decay (39). In addition, the rapid traverse of the percolation water could also reduce the soil leaching, contributing to a further decrease in the (^234^U/^238^U)_I_ (40).

## Discussion

### Comparisons of Stalagmite Geochemical Records in CA.

Several Holocene stalagmite records from CA have been reported. Cheng et al. (41) presented a stalagmite δ^18^O record from Kesang cave in Xinjiang, China, covering most of the past 500 ka. It shows similar patterns with the stalagmite δ^18^O records from the Asian monsoon region on orbital timescale with lower δ^18^O values during periods of high Northern Hemisphere summer insolation (NHSI) and vice versa (41). During the middle and late Holocene, the δ^18^O records from Kesang cave show a gradually increasing trend following the decreasing NHSI (25, 41). Recent stalagmite records from Baluk cave in Xinjiang, China (13) and Tonnel’naya cave in Uzbekistan (26) also show a positive δ^18^O trend since the middle Holocene. Our δ^18^O record is consistent with the existing stalagmite records from CA on orbital timescales. However, compared to existing data, our higher resolution record reveals more detailed information on centennial to decadal variability (*SI Appendix*, Fig. S7). The widely regional coherent variations of the stalagmite δ^18^O over CA indicate its control by large-scale atmospheric circulation (13, 26). In addition to δ^18^O changes, the stalagmite trace elements vary in different areas of CA. For example, the Sr/Ca records from Talisman cave and Tonnel’naya cave (26) in the WCA show long-term increasing trends, similar to their δ^18^O trends. However, decreasing trends are observed in the Sr/Ca records of stalagmites from Baluk (13) and Kesang cave (26, 41) in ECA (*SI Appendix*, Fig. S7). The decreasing trends are also evident in the Mg/Ca, Ba/Ca, and U/Ca ratios of the Baluk stalagmite (13). Those stalagmite trace element compositions are suggested as recording local precipitation/effective precipitation changes (13, 20, 26). The regional differences in the relationship between stalagmite trace elements and δ^18^O variations reveal that the “amount effect” is not the dominating factor controlling the precipitation/stalagmite δ^18^O changes in ECA, as recognized previously (13, 26).

Nevertheless, it does not exclude the possibility of speleothem δ^18^O being representative of precipitation amount in WCA. As shown in Fig. 2, the δ^18^O of F11 is positively correlated with other hydroclimatic proxies, such as δ^13^C, Sr/Ca ratios, and δ^234^U_initial_. It significantly loads on PC1 of F11 (*SI Appendix*, Table S4). This inverse δ^18^O-precipitation amount relationship in WCA is supported by model simulation results. Simulated data from an isotope-enabled general circulation model, ECHAM5-wiso (42), show significant negative correlations between the amount-weighted δ^18^O_p_ data extracted from the grid point closest to Talisman cave and both local and upstream (WCA, west Asia, and the Middle East) regional precipitation amount during 1950 to 2015 AD (Fig. 1). This negative relationship is supported by previous speleothem studies from West Asia (43) and the Middle East (44, 45). Strong WJ can cause enhanced Mediterranean storm tracks and colder winter/spring, bringing more precipitation in the Middle East, west Asia, and WCA (15, 20, 46), resulting in depleted δ^18^O_p_ of this region. In contrast, positive correlations are observed between simulated δ^18^O_p_ and precipitation amount in ECA, which is consistent with the observed inverse trend of stalagmite δ^18^O and trace elements in this region since the middle Holocene (Fig. 1).

### Orbital-driven Contrasting Precipitation Trends between WCA and ECA.

**Fig. 3.**
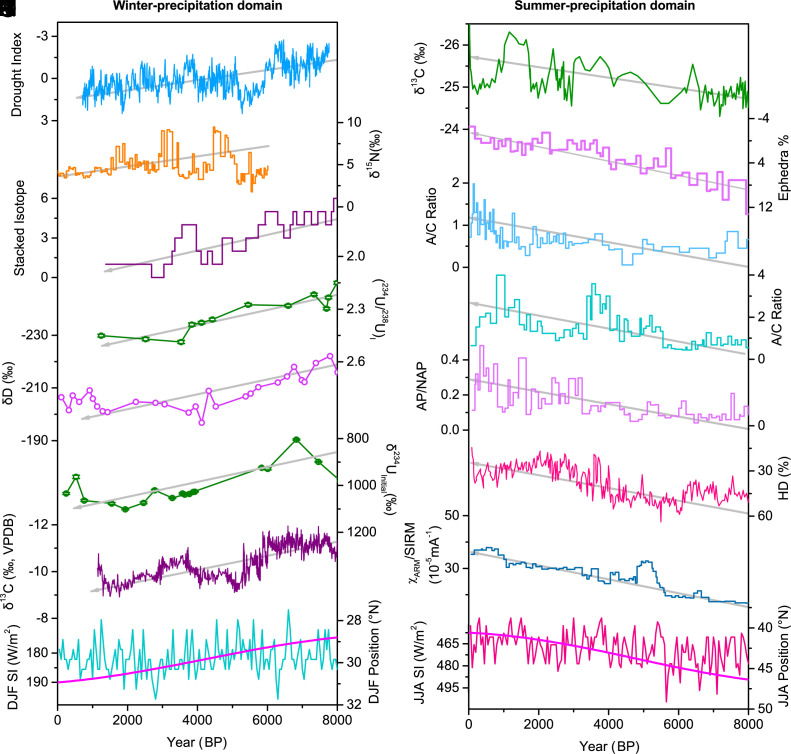
Comparisons of paleoclimate records from winter-precipitation and summer-precipitation domains and simulated winter and summer Westerly jet (WJ) positions in central Asia over the past 8,000 y. (*A*) drought index record from Talisman cave (this study); (*B*) δ^15^N record from Lake Son Kol in central Kyrgyzstan (28); (*C*) synthesized δ^18^O record (normalized) of six lakes from western Iran to the East Mediterranean region (49); (*D*) stalagmite (^234^U/^238^U)_I_ record from Katalekhor cave in northwestern Iran (30); (*E*) δD from Lake Neor in Northwestern Iran (29). (*F*) stalagmite δ^234^Uinitial records from Sofular cave in Turkey (32); (*G*) stalagmite δ^13^C from Jeita cave in Lebanon (31). (*H*) CESM simulated winter (December-January-February) WJ positions in central Asia (50); (*I*) δ^13^C record of Chaiwopu peat in Xinjiang, China (7); (*J*) sediment Ephedra percentage from Lake Bosten in Xinjiang, China (22); (*K*) sedimentary A/C ratios from Lake Aibi in Xinjiang, China (24); (*L*) sediment A/C ratios from Lake Sayram in Xinjiang, China (27); (*M*) arboreal/nonarboreal pollen (AP/NAP) ratios of Narenxia peat in Xinjiang, China (9); (*N*) humification degree of Tielishahan peat in Xinjiang, China (23) (*O*) magnetic index in LJW10 loess profile from Xinjiang, China (5); (*P*) CESM simulated summer (June-July-August) WJ positions in central Asia (50). The purple lines in panels H and P represent the winter and summer solar insolation (SI) at 40°N (51), respectively. The gray lines with arrows denote the long-term linear trends of the above paleoclimate records.

Our stalagmite record indicates a decreasing precipitation trend since the middle Holocene in WCA. A regionally wetter middle Holocene is suggested in archeological records. The number of archaeological sites increased in WCA, including today’s desert areas, during the middle Holocene (47, 48), while they significantly reduced after 6,000 y BP (20). This long-term decreasing precipitation trend in WCA may have extended to the winter precipitation–dominated West Asia and the East Mediterranean (Fig. 3). Roberts et al. (49) synthesized six lakes δ^18^O records extending from western Iran to the east Mediterranean (Fig. 3*C*), which reveal a shift to more positive δ^18^O values since 6,000 y BP (note the record shown in Fig. 3*C* was transformed as standardized normal values), indicating the drying trend. The orbital drying trend is further supported by recent, more precisely dated stalagmite and peat records. The increasing δ^18^O and (^234^U/^238^U)_I_, decreasing diameters of a stalagmite from northwestern Iran (Fig. 3*D*), reveal a progressive reduction of winter precipitation since the middle Holocene (30). The δD values of C28 n-alkanoic acid from a peat core in northwestern Iran also show a gradual increase over the last 8,000 y, indicating the long-term drying trend (Fig. 3*E*), which is in agreement with the enhanced aeolian input as recorded by elemental abundances (29). A stalagmite from the southern Black Sea coast also recorded increasing δ^234^U_initial_ values (Fig. 3*F*) and decreasing diameters since the middle Holocene, suggesting the drying trend (32). Furthermore, the drying trend was recorded in both the δ^13^C and Sr/Ca ratios of a stalagmite from the northern Levant (Fig. 3*G*) (31). These geological and biological records confirm the simulated increasing precipitation in the winter-precipitation domain during periods with perihelion at the Northern Hemisphere summer solstice and aphelion at the Northern Hemisphere winter solstice (15).

In contrast, the precipitation in ECA has increased since the middle Holocene (Fig. 3 *I*−*O*), which has been recognized in many studies (5, 7–10, 12, 20, 24, 52). As shown in Fig. 3*I*, the δ^13^C values of a well-dated peat core from central Tianshan show negative shifts over the past 8,000 y, indicating a steadily increasing trend of rainfall (7). During this period, the degree of humification of peat from the Altai Mountains shows a decreasing trend (Fig. 3*N*), suggesting weakening microbial activities under anaerobic reducing conditions caused by enhanced precipitation (23). The multiple environmental magnetic parameters (Fig. 3*O*) and grain size of loess-paleosol sequences in Xinjiang, China, confirm a persistent Holocene wetting trend (5). This wetting trend in ECA is also well-expressed in pollen records of lacustrine sediments (Fig. 3 *J*−*M*), such as in Ephedra percentage of Lake Bosten (22), A/C ratios of Lake Aibi (24) and Lake Sayram (27), and arboreal/nonarboreal pollen (AP/NAP) ratios of Narenxia peat bog (9).

We ascribe the orbital-driven contrasting precipitation trends between WCA and ECA to their different seasonal precipitation patterns. It is suggested that the precipitation in CA is dominated by the intensity and positions of the WJ (8, 46). A recent simulation study based on the Community Earth System Model (CESM) reveals a gradually strengthened and southward shift of the summer WJ over CA during the last 8,000 y (Fig. 3*P* and *SI Appendix*, Fig. S8*A*), which was controlled by the reduced NHSI. In contrast, the winter WJ shows a northward shift trend over the past 8,000 y (Fig. 3*H* and *SI Appendix*, Fig. S8*B*), following the increased Northern Hemisphere winter insolation (NHWI) (50). The southward and strengthened summer WJ enhanced the frequency and intensity of Mediterranean storms and brought more water vapor to inland CA, resulting in the wetting trend of the summer rainfall–dominated ECA over the past 8,000 y. However, the increase in summer precipitation did not significantly contribute to the annual precipitation of WCA, which is dominated by the amount of winter-half-year precipitation. On the contrary, the warming (53) and a northward shift of the WJ (50) in winter since the middle Holocene could reduce the Mediterranean storms, decrease the moisture transfer to CA, and result in reduced rainfall and snowfall in WCA during this period. This mechanism implies a possible long-term decreasing precipitation trend in WCA and West Asia under increasing NHWI and warming winters in the future.

This orbitally driven antiphased relationship between summer and winter precipitation in CA was well recorded in the sediment of Lake Son Kol in central Kyrgyzstan (28). The lake is located in today’s summer rainfall–dominated region but with a non-negligible portion of winter precipitation (Fig. 1). The total amount of n-alkanes n-C17-31 of the Son Kol sediment showed an increasing trend, and the δD_n-C29_ demonstrated a decreasing trend over the past 5,000 y, which indicates the increasing higher terrestrial plant abundance with wetting summer conditions. However, the decreasing δ^15^N suggested reduced terrestrial organic material input over the past 5,000 y, which resulted from decreased precipitation and/or meltwater runoff during winter/spring (Fig. 3*B*). It should be noted that the magnetic parameters and stable carbon isotope composition of bulk organic matter (δ^13^C_org_) from two loess-palaeosol sections from southeastern Uzbekistan (54) and northeastern Iran (55), respectively, reflect increasing soil moisture during the Holocene. However, this is not contradictory to the declining precipitation in the WCA. Considering the limited available precipitation in CA, temperature is the dominant controlling factor of soil formation. Decreasing summer temperature during the Holocene would have decreased the effective evaporation and improved the soil moisture availability. Together with the increasing winter temperature, it could enhance vegetation and soil development during the growing season (56) in the late Holocene, and control the soil-related proxy changes, such as δ^13^C_org_ and magnetic susceptibility. In contrast, higher summer temperatures and lower winter temperatures during the middle Holocene could suppress soil moisture and vegetation development in WCA, even if the precipitation in WCA was higher during this period than in the late Holocene.

### Centennial- to Decadal-scale Precipitation Variability in WCA.

**Fig. 4.**
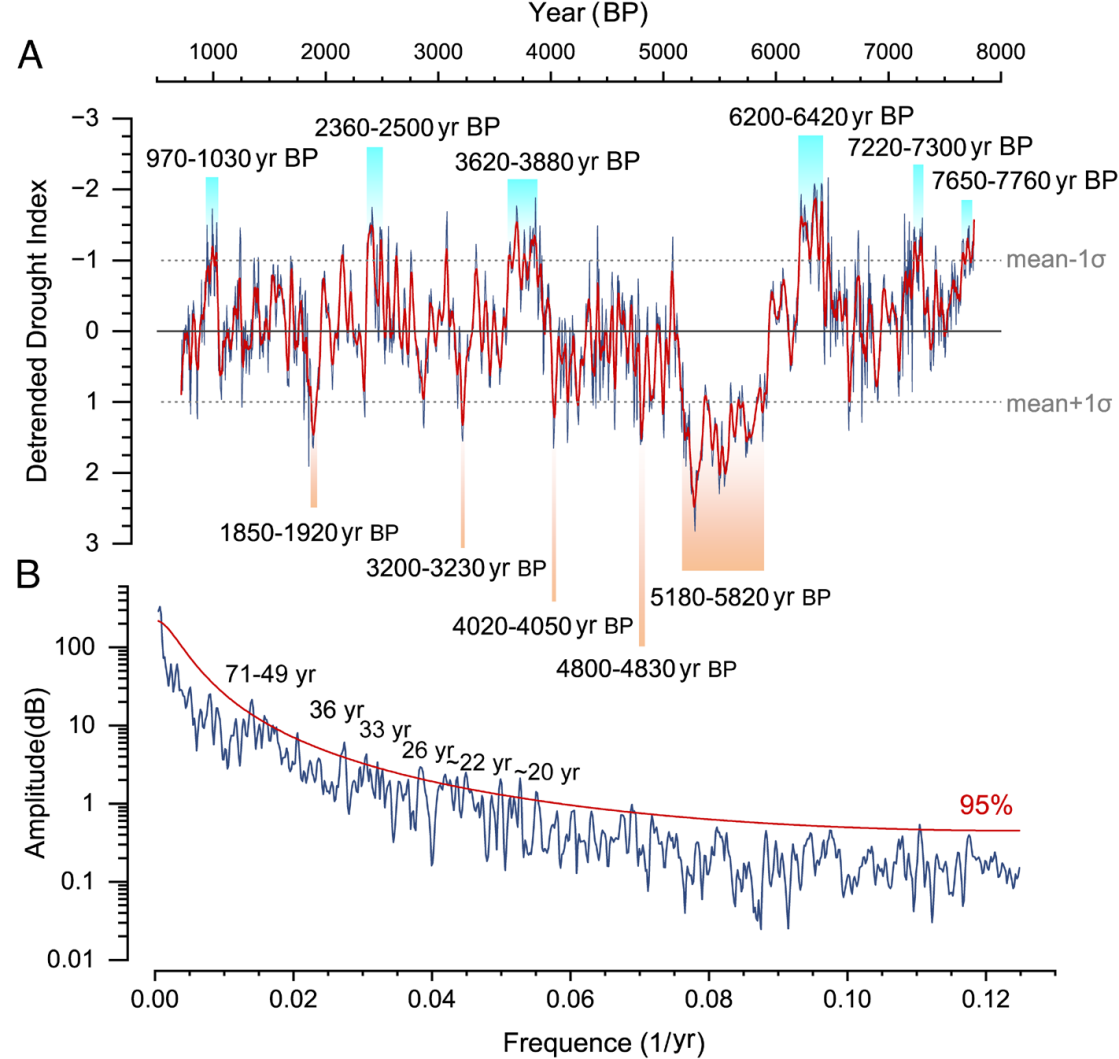
(*A*) Centennial- to-decadal-scale precipitation changes in WCA since 7,800 y BP as revealed by the detrended drought index record from Talisman cave. The red line is seven-point smoothed. Five droughts and six pluvials, which exceed 1σ of the whole series and last more than 30 y, are marked by orange and blue bars, respectively. (*B*) Spectral analysis of the drought index record during the past 8,000 y from Redfit 35 software (60). The parameters of the software used in this study were nsim = 1,000, mctest = T, rhopre = −99.0, ofac = 2, n50 = 4, and iwin = 1.

Ensemble empirical mode decomposition analysis suggests a dominant ~1,400 y periodicity in the precipitation variations in WCA, which explains the maximum variance (23%) of the drought index record (*SI Appendix*, Fig. S9). This periodicity is similar to the ~1,500 y climate cycle in the North Atlantic during the Holocene as recorded by proxies of drift ice in deep-sea sediment cores (57). Indeed, dry periods in WCA corresponded well with periods of increased storminess in the North Atlantic (*SI Appendix*, Fig. S10), indicating a northward shift of the westerly jet, akin to a present-day positive North Atlantic Oscillation (58). After removing the long-term linear trends from the drought index record, significant multidecadal quasiperiodicities of 20 to 30 and 50 to 70 y are evident (Fig. 4 and *SI Appendix*, Fig. S11), which are consistent with the North Atlantic Oscillation’s periodicities in a 5,200-y lake sediment record from southwestern Greenland (59).

Several centennial-to-decadal-scale droughts and pluvials are identified, as defined by values exceeding one SD (1σ) relative to data from the entire series. Five droughts were recorded in the intervals of 5,180 to 5,820, 4,800 to 4,830, 4,020 to 4,050, 3,200 to 3,230, and 1,850 to 1,920 yr BP. Six pluvial periods occurred in 7,650 to 7,760, 7,220 to 7,300, 6,200 to 6,420, 3,620 to 3,880, 2,360 to 2500, and 970 to 1,030 y BP (Fig. 4). As an intercontinental region, changes in CA climate have played an important role in regional societies and trans-Eurasian cultural exchange during the past several millennia. For example, the megadrought between 5,180 and 5,820 y BP was suggested to have impeded the culture expansion in CA and delayed the cultural exchange along the proto-Silk Road (20). After the megadrought, the gradually recovered precipitation promoted the flourishing of the Bronze Age civilization in WCA (Bactria–Margiana Archaeological Complex, ~4250 to 3,650 y BP or ~4,350 to 3,550 y BP) (61), and the southward migration of pastoralists from the Eurasian steppe (62, 63). The abrupt drying from 3,620 y BP after the pluvial period, together with the invasion of nomads, may have contributed to the rapid collapse of the Bactria–Margiana Archaeological Complex. In addition, the expansion of the Persian Empire (550 BC to 330 BC) corresponded well with the pluvial period in 2,360 to 2,500 BP (550 BC to 410 BC) in WCA, and probably in western Asia. More studies in the future could help to reveal the regional human–environment interactions and their impacts on the trans-Eurasian culture exchanges during historical and prehistorical times.

## Conclusions

Multiple geochemical proxies (δ^18^O, δ^13^C, Sr/Ca) in a stalagmite from the Fergana Valley, Kyrgyzstan, reveal precipitation variability in WCA on a wide range of timescales since 7,800 y BP. Different from the increasing precipitation trend in ECA since the middle Holocene, our synthesized precipitation record shows a long-term decreasing trend. The orbitally driven antiphased precipitation relationship between WCA and ECA resulted from different seasonal precipitation patterns, which were controlled by seasonal WJ changes. We propose that the long-term northward shift of the winter WJ under increasing NHWI during the Holocene could have reduced the Mediterranean storms, decreasing the moisture transfer to CA and resulting in the declining rainfall and snowfall in the winter precipitation–dominated WCA. In contrast, the southward and strengthened summer WJ, following the decreasing NHSI, may have caused the wetting trend of the summer precipitation–dominated ECA from the middle Holocene. We further identified a series of droughts and pluvial events on centennial- to decadal- timescales in WCA since 7,800 y BP, which may have influenced the regional societies and the trans-Eurasian cultural exchanges during historical and prehistorical times. Our findings provide, so far, the temporally most resolved climatic context for understanding the long history of culture change in the WCA. The orbital contrasting precipitation trends between WCA and ECA revealed in this study could help to improve the model projection of the future climate change in this eco-fragile region.

## Materials and Methods

### Chronology.

The chronologies of F11 and F2 are based on 33 and 16 ^230^Th dates, respectively. Each subsample was drilled parallel to the growth layer of the stalagmite in the polished section. The powders were dissolved in ultrapure nitric acid and followed the procedures described in Edwards et al. (64) to separate U and Th. Their concentration and isotopes were measured at a multicollector inductively coupled plasma mass spectrometer, Thermo Fisher Neptune, at the Isotope Laboratory, Xi’an Jiaotong University (65). All dates of F11 and F2 are in stratigraphic order (*SI Appendix*, Table S2). The age models and associated age uncertainties were modeled using COPRA age-modeling schemes (*SI Appendix*, Fig. S4) (66).

### Geochemical Proxy Analyses.

Subsamples for δ^18^O and δ^13^C analyses were milled at intervals of 0.1 and 0.15 mm for F11 and F2, respectively, by using a NEWWAVE Micromill device. All the subsamples were analyzed on an Elementar Isoprime100 isotope ratio mass spectrometer equipped with a MultiPrep system at the Institute of Earth Environment, Chinese Academy of Sciences, Xi’an. Standard NBS19 and TB1 were analyzed every 10 to 15 subsamples to check data reproducibility. The precision of measurements is better than 0.1‰ for both δ^18^O and δ^13^C with 2σ analytical errors. We scanned the Sr and Ca counts on the polished section of F11 at 0.1 mm interval, using a 4^th^-generation Avaatech X-ray fluorescence (XRF) core scanner equipped with the latest variable optical XRF technology following the method described in Li et al. (67). The analyses were carried out at the Institute of Earth Environment, Chinese Academy of Sciences. The average resolutions of the δ^18^O, δ^13^C, and Sr/Ca records of F11 are ~3.6-y and are ~4.4-y for the δ^18^O, δ^13^C records of F2 (Fig. 2 and *SI Appendix*, Fig. S5).

### Construction of Drought Index.

We linearly interpolated the δ^13^C, δ^18^O, and Sr/Ca record of F11 with a 4-y interval to not exceed the number of data points in the original time series. The PCA method was then applied to the interpolated geochemical proxy series. This method is a multivariate statistical data mining method used to reduce data size and highlight their similarities and differences. By using PCA, a large number of variables can be reduced to significantly important factors (68). Because precipitation was the common controlling factor of the δ^13^C, δ^18^O, and Sr/Ca ratios of F11, the first principal component, which explains 61% of the observed variance, was then used as a drought index in this region. Higher values indicate drier conditions and vice versa.

## Supplementary Material

Appendix 01 (PDF)

Dataset S01 (XLSX)

## Data Availability

All study data are included in the article and/or supporting information.
