# Supplementary material for: Hydroclimatic changes on multiple timescales since 7800 y BP in the winter precipitation–dominated Central Asia
Source: Proc Natl Acad Sci U S A. 2024 Mar 25;121(14):e2321645121. doi: 10.1073/pnas.2321645121 (PMC10998633; doi:10.1073/pnas.2321645121)
Supplement: Supplementary file 1 — Appendix 01 (PDF) [file pnas.2321645121.sapp.pdf]

PNAS

Supporting Information for

Hydroclimatic changes on multiple timescales since 7800 y BP in the winter precipitation–dominated Central Asia

Liangcheng Tan[a,b1], Hai Cheng[b,a], Dong Li[a,c], Rustam Orozbaev[d,e], Yanzhen Li[a], Hai Xu[f], R. Lawrence Edwards[g], Yougui Song[a], Le Ma[a], Fangyuan Lin[a], Ashish Sinha[h], Zhisheng An[a,i1]

[a]State Key Laboratory of Loess, Institute of Earth Environment, Chinese Academy of Sciences, Xi'an 710061, China;
[b]Institute of Global Environmental Change, Xi'an Jiaotong University, Xi'an 710054, China;
[c]Library of Chang'an University, Xi'an 710064, China;
[d]Research Center for Ecology and Environment of Central Asia (Bishkek), Chinese Academy of Sciences, Bishkek 720040, Kyrgyzstan;
[e]Institute of Geology, National Academy of Sciences of Kyrgyz Republic, Bishkek 720040, Kyrgyzstan;
[f]Institute of Surface-Earth System Science, Tianjin University, Tianjin 300072, China;
[g]Department of Earth and Environmental Sciences, University of Minnesota, Minneapolis, MN 55455;
[h]Department of Earth Science, California State University, Dominguez Hills, Carson, CA 90747;
[i]Interdisciplinary Research Center of Earth Science Frontier, Beijing Normal University, Beijing 100875, China.

[1]Corresponding authors: Zhisheng An: anzs@loess.llqg.ac.cn;
or, Liangcheng Tan: tanlch@ieecas.cn.

This PDF file includes:

Figures S1 to S11
Tables S1 to S4
SI References

Figures and Tables

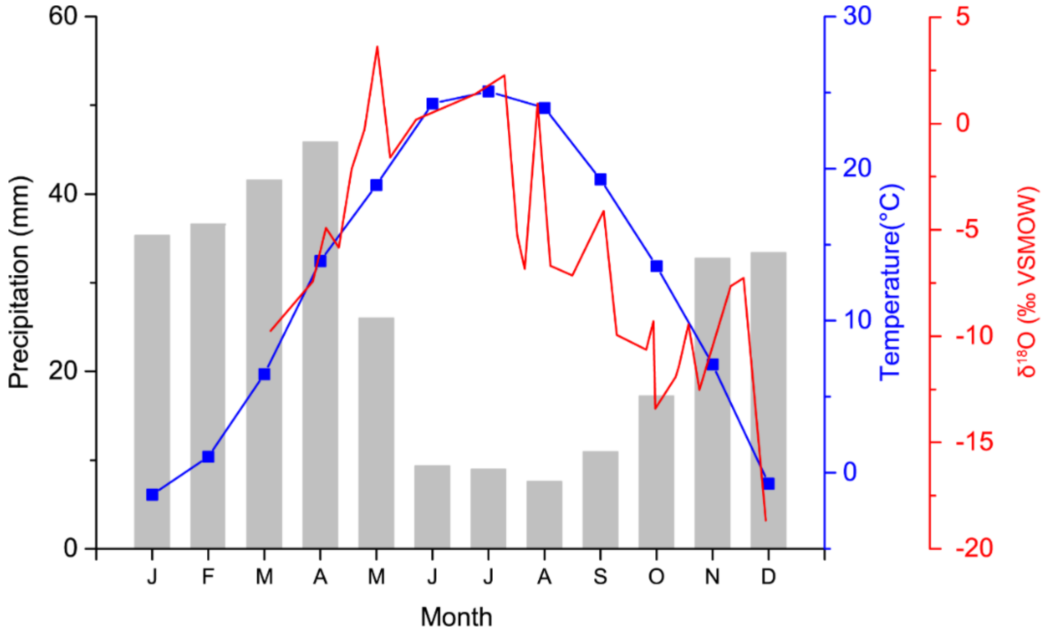

Figure S1. Annual mean temperature (~13 °C) and precipitation (~300 mm) in the area of Talisman Cave. The highest temperature occurs in July (25 °C), and lowest in January (-1.4°C) (data from meteorological station at Osh, 40 km east of Talisman Cave). Observed data from the Bishkek station (300 km northeast of Osh) show lower precipitation $\delta^{18}O$ during winter and spring than that in summer and autumn.

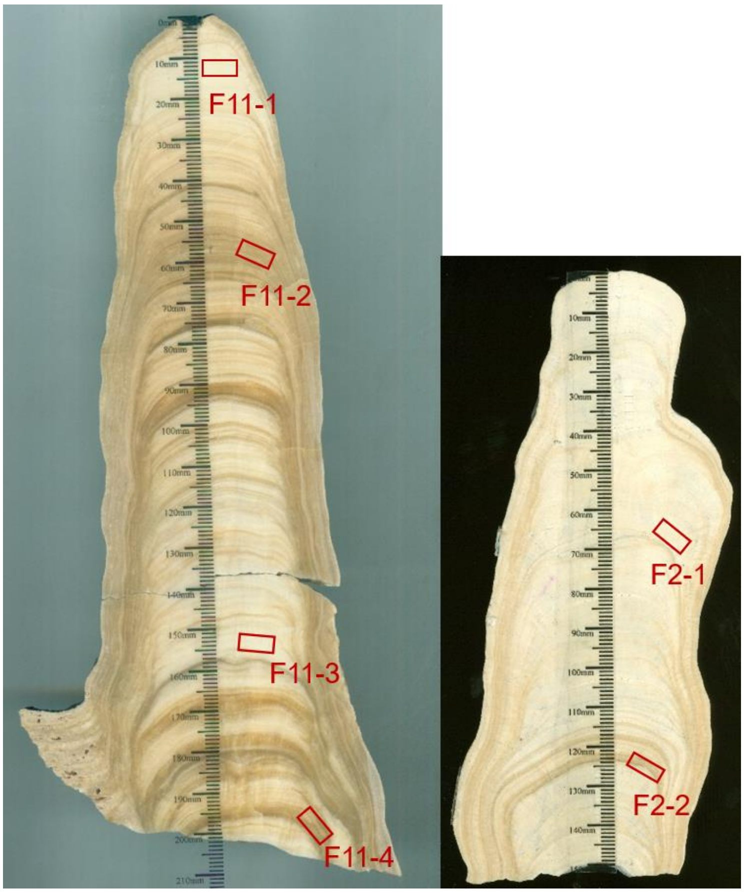

Figure S2. Polished section of stalagmite F11 and F2. The total lengths of F11 and F2 are 20 and 15 cm, respectively. Red boxes mark the locations of XRD analyses.

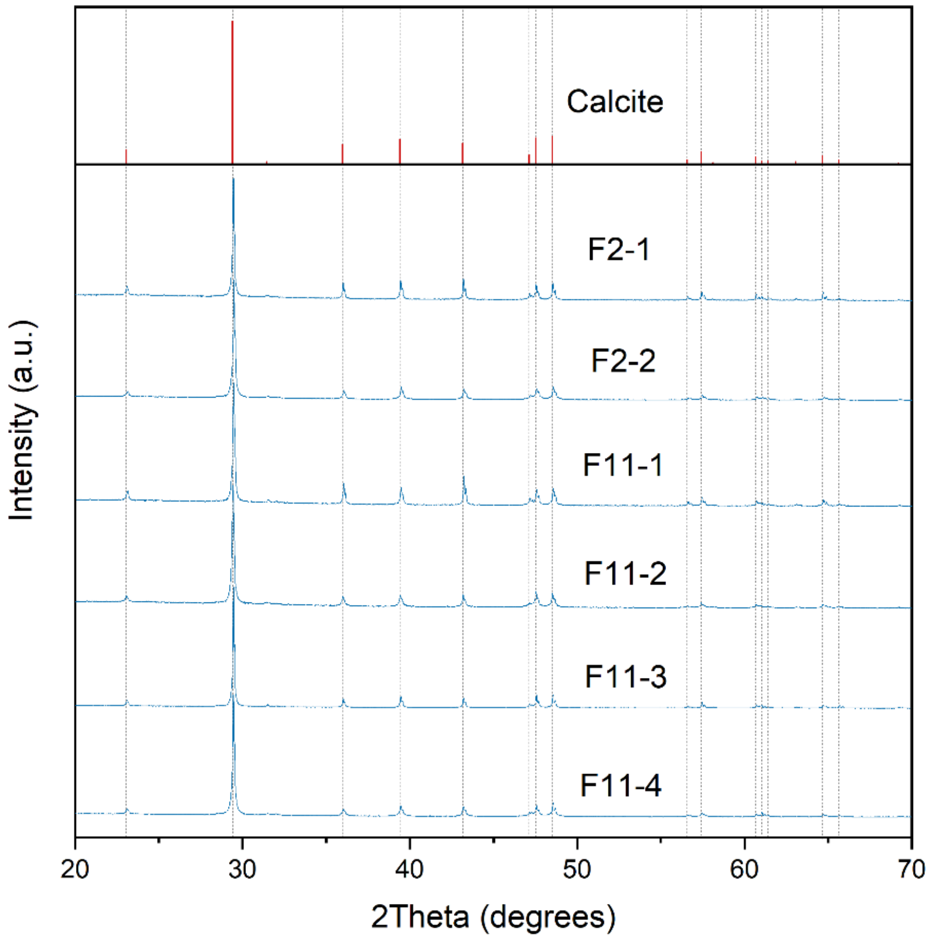

Figure S3. Results of XRD analyses for F2 and F11. F2-1, F11-1, and F11-3 were drilled from light layers, and the other three sub-samples (F2-2, F11-2, F11-4) were drilled from dark layers, with F2-2 from the hiatus. Results indicate that F2 and F11 are composed of calcite.

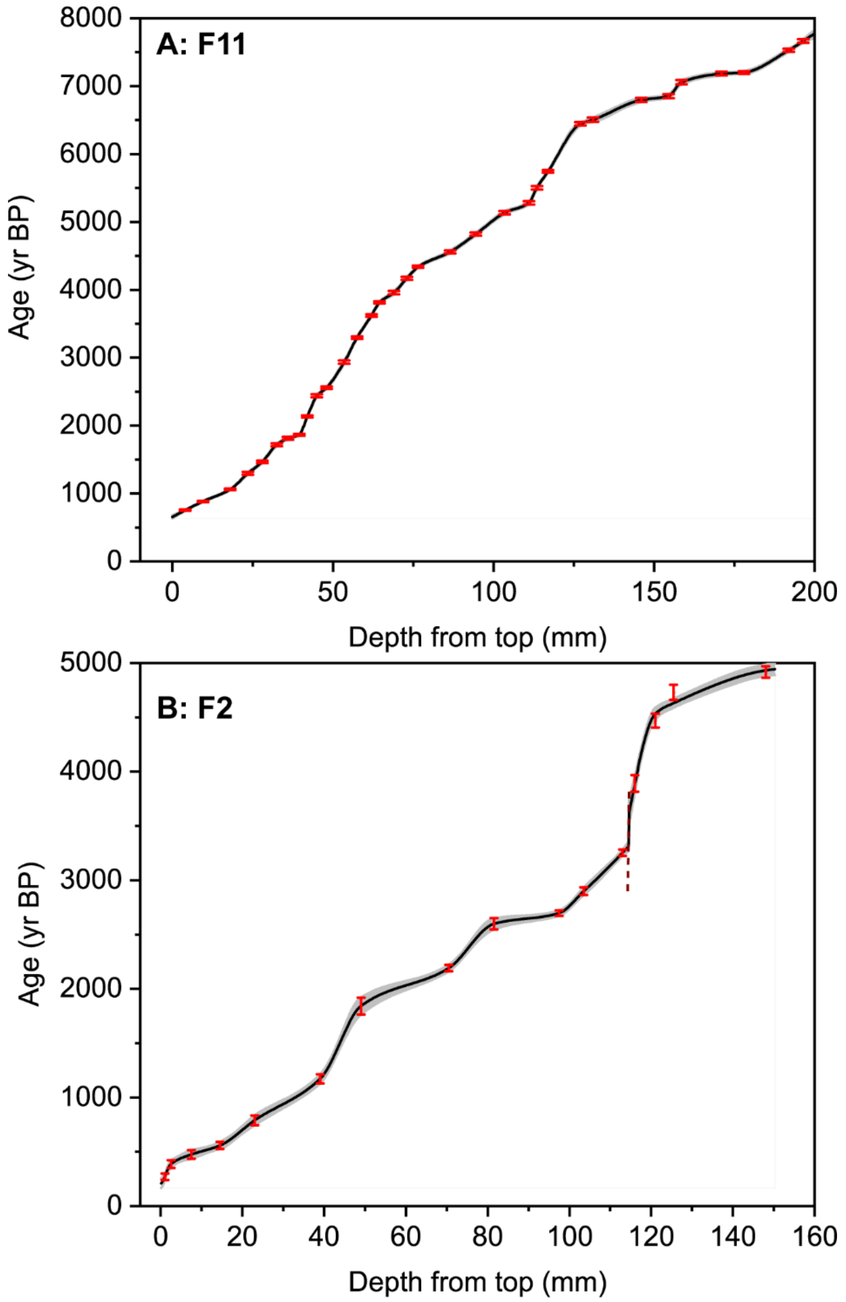

Figure S4. Age-depth models of (A) F11 and (B) F2. Age models were established using 5000 Monte-Carlo simulations in the COPRA routine (ref. 66). Gray lines represent the 95% confidence intervals and black lines represent the median ages, respectively. Error bars are 2σ error (red). Dash line in panel B denotes a hiatus of 114.5 mm from the top of the F2.

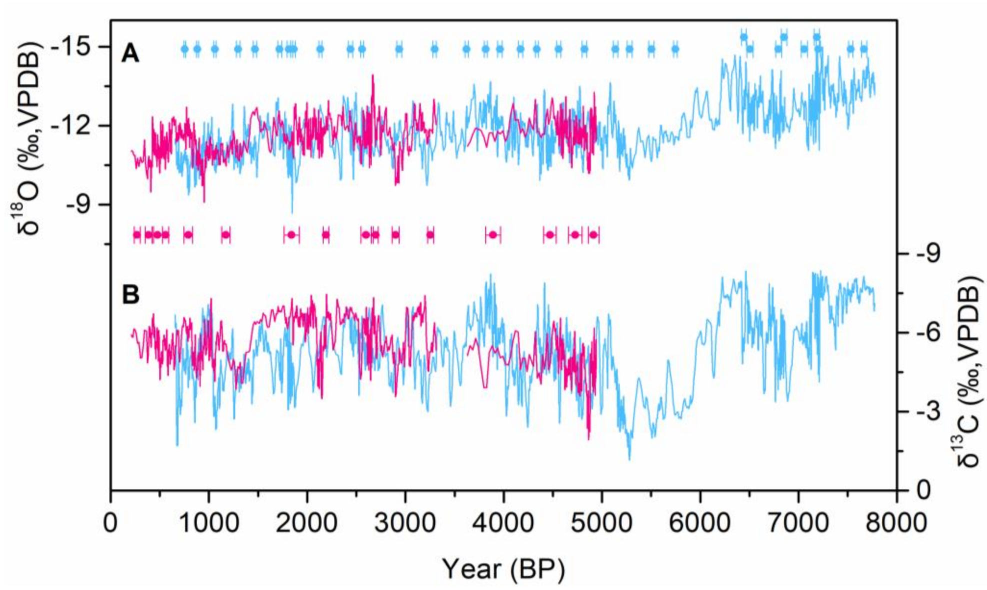

Figure S5. Comparisons of the (A) $\delta^{18}O$ and (B) $\delta^{13}C$ records between F11 (blue line) and F2 (purple line). Dots with error bars represent ^{230}Th dates of F11 and F2, respectively. Both the $\delta^{18}O$ and $\delta^{13}C$ records replicate well within the dating errors of the two stalagmites, indicating their deposition at or near isotopic equilibrium fractionation conditions (ref. 21).

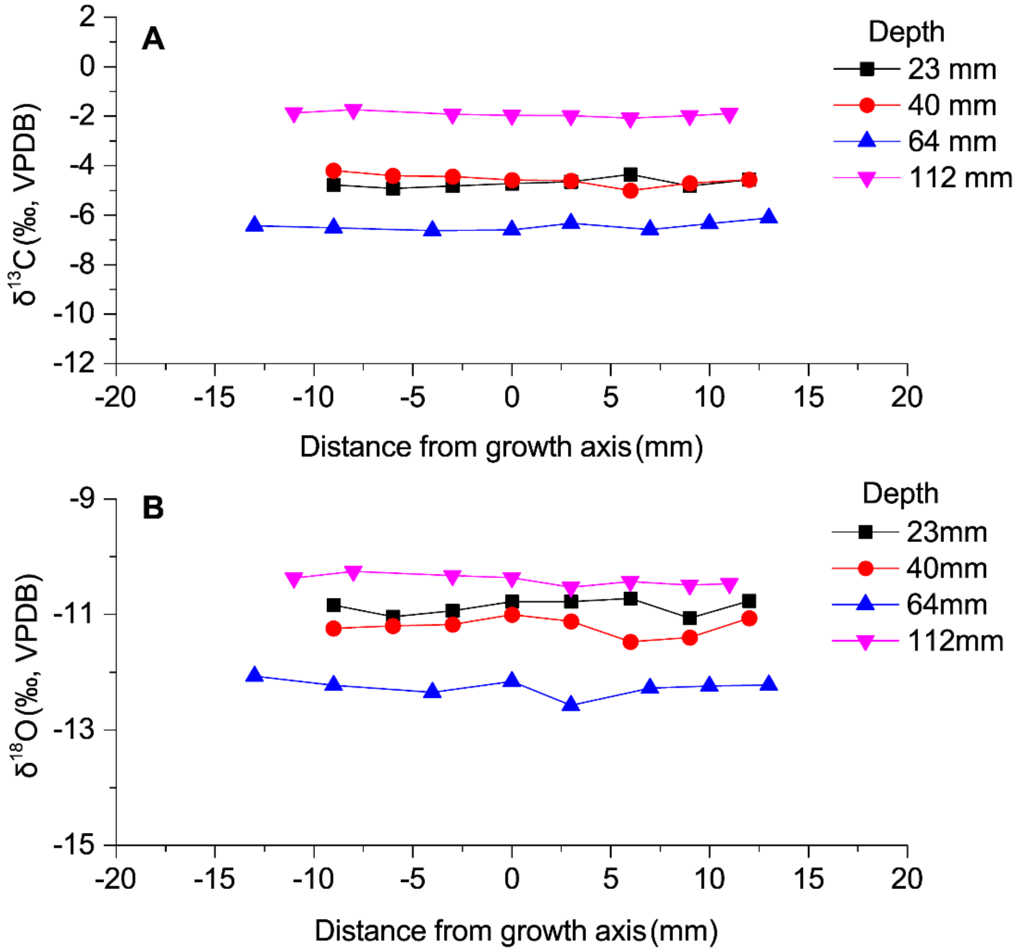

Figure S6. "Hendy test" on four layers of F11, with Panel A for carbon isotopes, and panel B for oxygen isotopes (ref. 20).

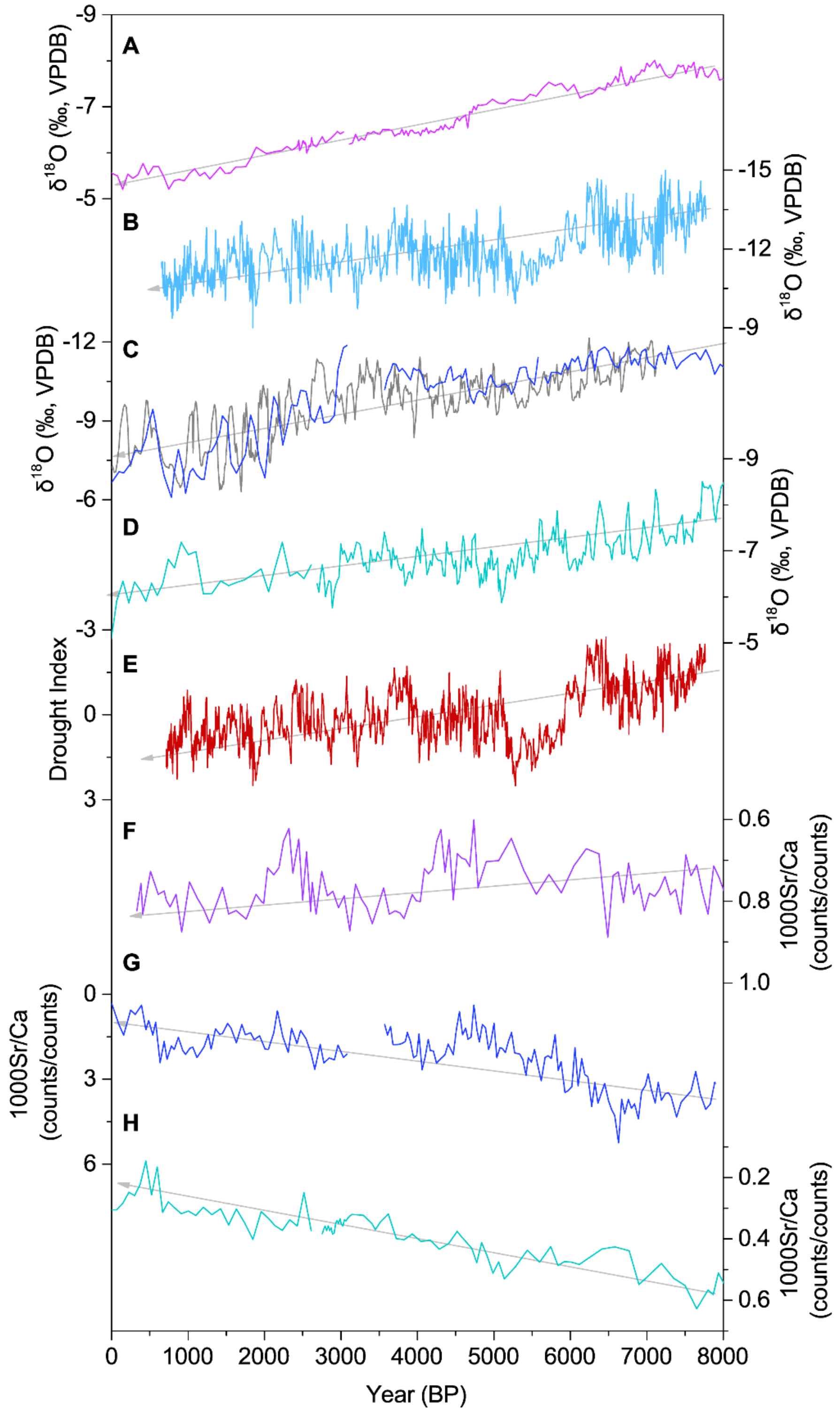

Figure S7. Comparisons of $\delta^{18}O$ and Sr/Ca ratio records of stalagmites from WCA and ECA during the past 8000 years. (A) $\delta^{18}O$ record from Ton cave (ref. 26); (B) $\delta^{18}O$ record from Talisman cave (this study); (C) $\delta^{18}O$ record from Kesang cave (ref. 25,26); (D) $\delta^{18}O$ record from Baluk cave (ref. 13); (E) Sr/Ca record from Talisman cave (this study); (F) Sr/Ca record from Ton cave (ref. 26); (G) Sr/Ca record from Kesang cave (ref. 26); (H) Sr/Ca record from Baluk cave (ref. 13). The grey lines with arrows represent the linear trends of the records, respectively.

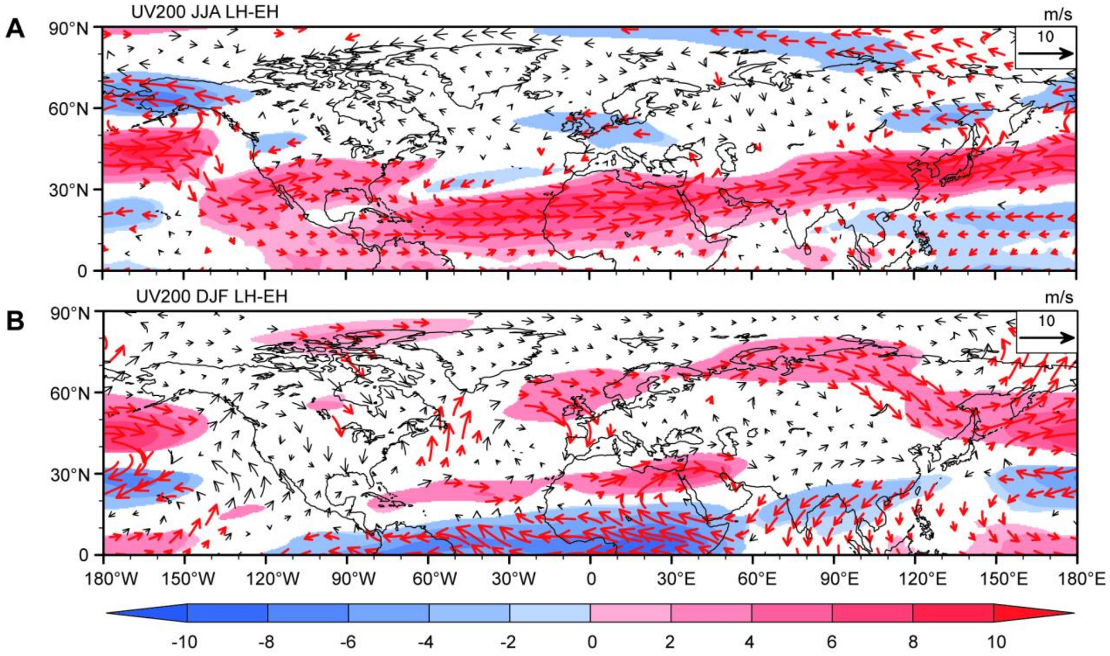

Figure S8. Summer (A) and winter (B) westerly jet differences between the late Holocene (LH) and early Holocene (EH) from the Community Earth System Model (CESM) simulation. The westerly jet variations are represented by the differences of 200 hPa wind field (m/s). Red vectors and shading denote the differences significant at the 95% confidence level (ref. 51).

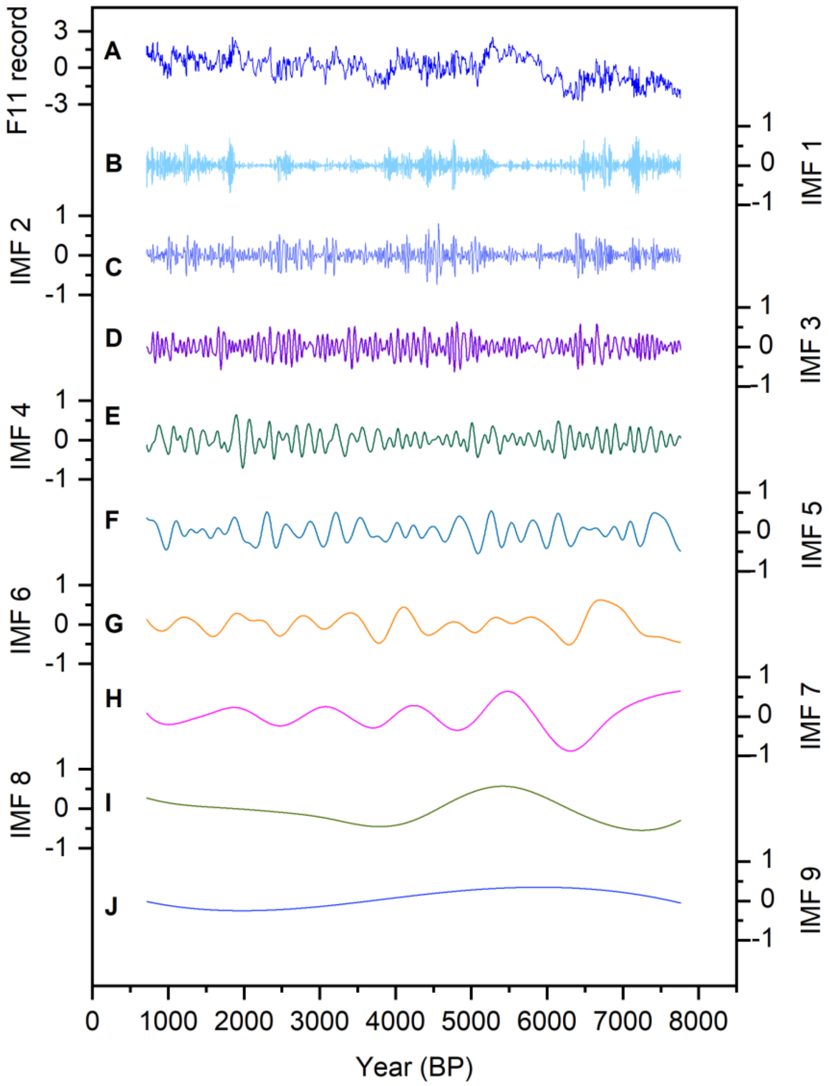

Figure S9. Intrinsic components of the (A) drought index record from ensemble empirical mode decomposition (EEMD) analysis. A white noise of 0.2 and an ensemble number of 100 were used for the EEMD decomposition (ref. S1). Nine EEMD components are presented in panels B to J. Components 1 through 7 represent quasi-oscillatory periodicities of 15, 32, 70, 142, 327, 829, and 1409 years, contributing 5%, 6%, 8%, 8%, 10%, 10%, 23% to the total variance of the drought index record, respectively. Components 8 and 9 represent long-term trends.

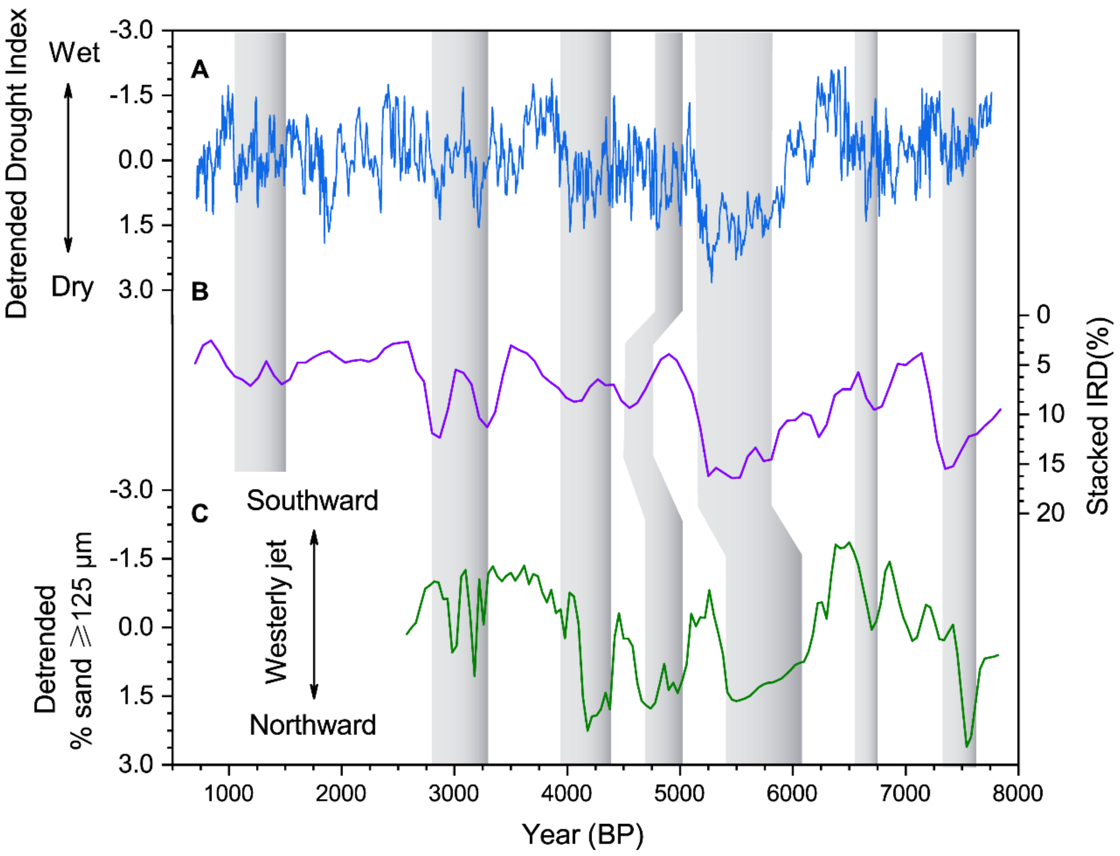

Figure S10. Comparisons of precipitation changes in WCA and climate changes in the North Atlantic since 7800 yr BP. (A) Detrended drought index record from WCA (this study); (B) hematite-stained grains record of Ice-Rafted Debris (IRD) in the North Atlantic (ref. 57); (C) storminess activities in the North Atlantic recorded by detrended coarse sand (>125 μm) percentage (ref. 58). The grey bars mark notable dry periods in WCA, which corresponded to increased storminess and IRD in the North Atlantic, suggesting northward shifts of the westerly jet.

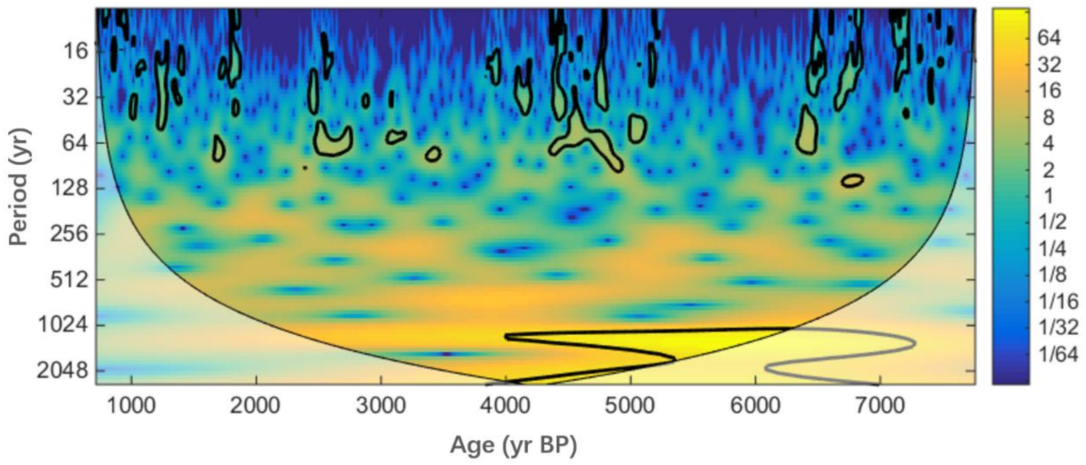

Figure S11. Continuous transform wavelet spectra for the drought index record of F11 from WCA. Spectral power is shown by colors ranging from deep blue (weak) to yellow (strong). Irregular black curves represent 95% confidence time-frequency regions in which the spectral strength is above that expected for an AR(1) red noise signal (ref. S2).

Table S1. Locations of sites referred in Figure 1

Site no.	Site name	Long. (°E)	Lat. (°N)	References
1	Chaiwopu peat	88.0	40.5	*Ref. 7*
2	Lake Bosten	87.0	42.0	*Ref. 22*
3	Tielishahan peat	86.9	48.8	*Ref. 23*
4	Narenxia peat	86.9	48.8	*Ref. 9*
5	Lujiaowan loess	85.3	44.0	*Ref. 5*
6	Baluk cave	84.7	42.4	*Ref. 13*
7	Lake Aibi	83.0	45.0	*Ref. 24*
8	Kesang cave	81.8	42.9	*Ref. 25,26*
9	Lake Sayram	81.2	44.6	*Ref. 27*
10	Lake Son Kol	75.1	41.8	*Ref. 28*
11	Talisman cave	72.4	40.4	*This study*
12	Ton cave	67.2	38.4	*Ref. 26*
13	Lake Neor	48.6	38.0	*Ref. 29*
14	Katalekhor cave	48.2	35.8	*Ref. 30*
15	Jeita cave	35.7	34.0	*Ref. 31*
16	Sofular cave	32.0	41.4	*Ref. 32*

Table S2. ^{230}Th dating results.

Sample Number	Depth (mm)	^{238}U (ppb)		^{232}Th (ppt)		^{230}Th / ^{232}Th (atomic x10^{-6})		d^{234}U* (measured)		^{230}Th / ^{238}U (activity)		^{230}Th Age (yr) (uncorrected)		^{230}Th Age (yr) (corrected)		δ$^{234}U_{Initial}$** (corrected)		^{230}Th Age (yr BP)*** (corrected)	
**F11-1**	4	637.9	±0.8	304	±7	421	±10	605.3	±1.9	0.0122	±0.0001	830	±6	821	±9	607	±2	**755**	**±9**
**F11-2**	9.5	606.4	±0.9	133	±4	1034	±29	583.3	±2.2	0.0138	±0.0001	954	±8	950	±9	585	±2	**884**	**±9**
**F11-3**	18	487.4	±0.6	280	±6	470	±11	578.6	±1.9	0.0164	±0.0001	1137	±9	1127	±11	580	±2	**1061**	**±11**
**F11-1a**	23.5	443.1	±0.3	392	±8	390	±9	659.4	±1.5	0.0209	±0.0002	1382	±15	1366	±19	662	±2	**1298**	**±19**
**F11-2a**	28	473.3	±0.5	142	±3	1286	±33	664.0	±1.8	0.0234	±0.0002	1542	±16	1536	±17	667	±2	**1468**	**±17**
**F11-3a**	32.5	395.4	±0.4	159	±4	1095	±28	630.2	±1.7	0.0266	±0.0003	1796	±19	1788	±20	633	±2	**1720**	**±20**
**F11-4a**	36	378.2	±0.3	235	±5	688	±15	502.4	±1.6	0.0259	±0.0002	1894	±17	1882	±19	505	±2	**1814**	**±19**
**F11-4**	39.5	433.6	±0.6	180	±4	1055	±26	503.8	±2.0	0.0265	±0.0002	1939	±12	1931	±13	507	±2	**1865**	**±13**
**F11-5a**	42	538.9	±0.5	361	±7	792	±17	600.7	±1.8	0.0322	±0.0002	2214	±14	2201	±16	604	±2	**2133**	**±16**
**F11-1c**	45	519.1	±0.7	182	±4	1784	±43	659.4	±1.8	0.0379	±0.0003	2514	±20	2508	±21	664	±2	**2440**	**±21**
**F11-6a**	48	528.3	±0.5	171	±4	1963	±46	611.8	±1.7	0.0385	±0.0002	2632	±16	2626	±16	616	±2	**2558**	**±16**
**F11-7a**	53.5	479.8	±0.5	697	±14	489	±10	567.6	±1.9	0.0430	±0.0002	3031	±16	3004	±25	572	±2	**2936**	**±25**
**F11-8a**	57.5	480.6	±0.5	177	±4	2228	±50	630.7	±1.9	0.0497	±0.0002	3370	±16	3364	±17	637	±2	**3296**	**±17**
**F11-9a**	62	550.7	±0.7	302	±6	1565	±33	555.4	±2.0	0.0520	±0.0002	3700	±14	3690	±16	561	±2	**3622**	**±16**
**F11-6**	64.5	528.2	±0.7	257	±5	1788	±38	500.0	±1.9	0.0527	±0.0002	3892	±14	3882	±16	506	±2	**3816**	**±16**
**F11-10a**	69	481.5	±0.5	296	±6	1424	±31	458.4	±1.7	0.0531	±0.0003	4041	±22	4028	±24	464	±2	**3960**	**±24**
**F11-11a**	73	520.0	±0.5	425	±9	1196	±25	547.0	±1.5	0.0593	±0.0002	4253	±18	4238	±21	554	±1	**4170**	**±21**
**F11-7**	76.5	430.9	±0.6	100	±2	4294	±102	523.8	±2.1	0.0605	±0.0002	4408	±15	4404	±16	530	±2	**4338**	**±16**
**F11-8**	86.5	466.3	±0.6	174	±4	2843	±64	541.5	±2.0	0.0643	±0.0003	4633	±21	4626	±22	549	±2	**4560**	**±22**
**F11-9**	94.5	416.5	±0.6	135	±3	3539	±83	581.7	±2.1	0.0696	±0.0003	4894	±24	4888	±24	590	±2	**4822**	**±24**
**F11-12a**	103.5	436.8	±0.5	155	±4	3365	±80	544.1	±1.8	0.0723	±0.0003	5212	±23	5205	±24	552	±2	**5137**	**±24**
**F11-10**	111	367.0	±0.5	160	±4	2943	±66	617.2	±2.0	0.0777	±0.0003	5354	±24	5347	±25	627	±2	**5281**	**±25**
**F11-2c**	113.5	363.4	±0.3	167	±4	2812	±62	568.3	±1.5	0.0785	±0.0004	5580	±26	5571	±27	577	±2	**5503**	**±27**
**F11-13a**	117	490.9	±0.5	192	±4	3475	±75	582.1	±1.5	0.0825	±0.0002	5821	±17	5814	±18	592	±1	**5746**	**±18**
**F11-15a**	127	448.0	±0.5	137	±3	4437	±107	418.0	±1.6	0.0825	±0.0003	6517	±25	6511	±25	426	±2	**6443**	**±25**

Continue to next page

Sample Number	Depth (mm)	^{238}U (ppb)	^{232}Th (ppt)	^{230}Th / ^{232}Th (atomic x10^{-6})	d^{234}U* (measured)	^{230}Th / ^{238}U (activity)	^{230}Th Age (yr) (uncorrected)	^{230}Th Age (yr) (corrected)	$\delta^{234}U_{Initial}$** (corrected)	^{230}Th Age (yr BP)*** (corrected)
**F11-11**	131	373.7 ±0.5	156 ±4	3364 ±80	451.8 ±1.7	0.0852 ±0.0004	6579 ±32	6571 ±32	460 ±2	**6505 ±32**
**F11-16a**	146	375.0 ±0.4	76 ±2	6986 ±212	399.6 ±1.5	0.0857 ±0.0003	6869 ±29	6865 ±29	407 ±2	**6797 ±29**
**F11-12**	154.5	376.0 ±0.4	93 ±2	5908 ±160	436.9 ±1.4	0.0887 ±0.0004	6924 ±29	6919 ±29	445 ±1	**6853 ±29**
**F11-13**	158.5	327.7 ±0.3	225 ±5	2197 ±48	441.6 ±1.4	0.0916 ±0.0004	7138 ±32	7124 ±33	451 ±1	**7058 ±33**
**F11-14**	171	473.4 ±0.7	89 ±2	8387 ±227	481.4 ±2.0	0.0957 ±0.0003	7256 ±27	7252 ±27	491 ±2	**7186 ±27**
**F11-17a**	178	583.3 ±0.7	121 ±3	7615 ±181	477.4 ±1.8	0.0956 ±0.0002	7272 ±21	7268 ±21	487 ±2	**7200 ±21**
**F11-18a**	192	503.8 ±0.6	115 ±3	7305 ±181	498.1 ±1.8	0.1013 ±0.0003	7603 ±23	7599 ±23	509 ±2	**7531 ±23**
**F11-16**	196.5	470.9 ±0.7	96 ±2	8340 ±215	494.8 ±1.9	0.1027 ±0.0003	7736 ±27	7732 ±27	506 ±2	**7666 ±27**
**F2-1A**	1	754.9 ±1.9	1319 ±27	51 ±3	620.1 ±3.3	0.0054 ±0.0003	367 ±19	335 ±29	621 ±3	**270 ±29**
**F2-1**	2.5	624.6 ±1.6	1307 ±27	57 ±3	605.9 ±2.7	0.0072 ±0.0003	489 ±22	451 ±35	607 ±3	**386 ±35**
**F2-2A**	7.5	603.9 ±1.1	1430 ±29	60 ±3	621.6 ±2.4	0.0087 ±0.0004	584 ±25	541 ±39	623 ±2	**476 ±39**
**F2-3A**	14.5	593.0 ±1.1	814 ±17	114 ±5	599.3 ±2.3	0.0095 ±0.0004	648 ±27	623 ±33	600 ±2	**558 ±33**
**F2-4A**	23	527.2 ±1.0	1298 ±26	89 ±4	616.4 ±2.4	0.0133 ±0.0005	900 ±32	855 ±45	618 ±2	**790 ±45**
**F2-5A**	39	607.2 ±1.4	1568 ±32	121 ±4	622.0 ±2.9	0.0190 ±0.0004	1283 ±27	1237 ±42	624 ±3	**1172 ±42**
**F2-6A**	49	658.8 ±1.2	3755 ±76	85 ±2	612.3 ±2.4	0.0295 ±0.0004	2009 ±30	1906 ±78	616 ±2	**1841 ±78**
**F2-3**	70.5	557.3 ±1.5	784 ±16	406 ±9	672.7 ±3.1	0.0347 ±0.0004	2281 ±24	2256 ±30	677 ±3	**2191 ±30**
**F2-8A**	81.5	500.9 ±0.9	1866 ±38	188 ±4	715.9 ±2.6	0.0424 ±0.0004	2726 ±27	2663 ±52	721 ±3	**2598 ±52**
**F2-9A**	97.5	608.4 ±1.1	916 ±19	471 ±10	701.7 ±2.4	0.0430 ±0.0003	2788 ±19	2762 ±26	707 ±2	**2697 ±26**
**F2-10A**	103.5	570.9 ±0.8	1267 ±26	354 ±8	749.4 ±2.2	0.0476 ±0.0004	3001 ±23	2964 ±35	756 ±2	**2899 ±35**
**F2-11A**	113	699.3 ±1.0	1359 ±27	440 ±9	708.8 ±2.3	0.0518 ±0.0003	3351 ±20	3318 ±30	715 ±2	**3253 ±30**
**F2-2b**	116	593.4 ±0.7	3205 ±64	190 ±4	706.8 ±1.9	0.0623 ±0.0006	4048 ±39	3956 ±75	715 ±2	**3891 ±75**
**F2-4b**	121	813.4 ±0.9	1885 ±39	475 ±11	620.2 ±1.8	0.0668 ±0.0008	4577 ±57	4535 ±64	628 ±2	**4470 ±64**
**F2-12A**	125.5	574.6 ±0.8	3077 ±62	227 ±5	674.3 ±2.4	0.0736 ±0.0003	4887 ±23	4794 ±70	683 ±2	**4729 ±70**
**F2-2**	148	611.8 ±4.2	527 ±12	1524 ±35	772.2 ±8.2	0.0796 ±0.0007	4994 ±52	4980 ±53	783 ±8	**4915 ±53**

Analytical errors are 2s of the mean.
U decay constants: λ_{238} = 1.55125x10^{-10} (ref. S3) and λ_{234} = 2.82206x10^{-6} (ref. 65). Th decay constant: λ_{230} = 9.1705x10^{-6} (ref. 65).
*$\delta^{234}U$ = ([$^{234}U/^{238}U$]$_{activity}$ – 1)x1000. ** $\delta^{234}U_{initial}$ was calculated based on ^{230}Th age (T), i.e., $\delta^{234}U_{initial}$ = $\delta^{234}U_{measured}$ x $e^{\lambda 234 x T}$.
Corrected ^{230}Th ages assume the initial $^{230}Th/^{232}Th$ atomic ratio of 4.4 ±2.2 x10^{-6}. Those are the values for a material at secular equilibrium, with the bulk earth $^{232}Th/^{238}U$ value of 3.8. The errors are arbitrarily assumed to be 50%.
***B.P. stands for "Before Present" where the "Present" is defined as the year 1950 A.D.

Table S3. The Pearson correlation matrix of the $\delta^{18}O$, $\delta^{13}C$, and Sr/Ca data of F11. Asterisks (*) mean significance at 99% confidence level (two-tailed, t-test; after accounting for autocorrelation). All records are linearly interpolated to 4-yr resolution age-depth scale where appropriate before analysis.

	$\delta^{18}O$	$\delta^{13}C$	Sr/Ca
$\delta^{18}O$	-	0.706*	0.289*
$\delta^{13}C$		-	0.151*
Sr/Ca			-

*Correlation is significant at the 0.01 level (2-tailed)

Table S4. Component Matrix of the Principal Component Analysis for the $\delta^{18}O$, $\delta^{13}C$, and Sr/Ca records of F11. All records are linearly interpolated to 4-yr resolution prior to analysis.

	Component
	1*
$\delta^{18}O$	0.913
$\delta^{13}C$	0.869
Sr/Ca	0.481

*1 component extracted

SI References

1. Z. Wu, N. E. Huang, Ensemble empirical mode decomposition: a noise-assisted data analysis method. *Adv Adap Data Analy* **1**, 1-41 (2009).
2. C. Torrence, G. P. Compo, A Practical Guide to Wavelet Analysis. *Bull Amer Meteor Soc* **79**, 61-78 (1998).
3. A. Jaffey, K. Flynn, L. Glendenin, W. t. Bentley, A. Essling, Precision measurement of half-lives and specific activities of ^{235}U and ^{238}U. *Physical Review C***4**, 1889 (1971).
